# The Present and Future of Virtual Reality in Medical Education: A Narrative Review

**DOI:** 10.7759/cureus.51124

**Published:** 2023-12-26

**Authors:** Dipal Mistry, Callaham A Brock, Tom Lindsey

**Affiliations:** 1 Medicine, Edward Via College of Osteopathic Medicine (VCOM) Carolinas, Spartanburg, USA; 2 Simulation and Technology/Surgery, Edward Via College of Osteopathic Medicine (VCOM) Carolinas, Spartansburg, USA

**Keywords:** medical student simulation training, medical student teaching, behavioral empathy, simulation in medical education, medical student training, virtual reality, virtual reality simulation, realistic simulation

## Abstract

Virtual reality (VR) uses computer-generated and three-dimensional environments to create immersive experiences through the use of interactive devices that simulate virtual environments in many forms, such as 3D, screen-based, or room-based. Users can engage in the environment with objects, characters, and scenes, making individuals assume they are experiencing a real-life scenario. VR has been adopted across medical and nursing fields to supplement clinically relevant and practical teaching. However, the effectiveness of this interactive form of learning has come a long way with improvements in accessibility, cost, and technicalities. The immersive simulated environment that VR has to offer today initially began with screen-based learning and then the 360-video method. These previously sought-out methods were eventually found to disconnect the students from engaging in the learning environment that present-day VR systems are designed to provide. Interactive VR offers a dynamic platform for medical training. These simulations benefit the learner by allowing them to interact within case scenarios and virtual wards, as well as with patients, colleagues, and relatives. To mimic real-life encounters, the student can take a patient’s history and physical exam, investigate, diagnose, and provide treatment. The simulated patient can express emotions, concerns, and signs of a poor state of health. All these factors play into a healthcare provider’s competency to think critically and clinically in decision-making. This practice is now being used in many surgical programs and medical education curricula. The use of simulation in VR is continuously being proven to decrease injury, increase operation speed, and improve overall outcomes in patient-centered care. VR simulation differs from in-person simulation training in that the VR modality of learning is more accessible and replicable than the latter. By comparing research studies and reviews of medical programs that incorporated VR into their curricula, we were able to assess the state of VR in medical education and where this technology could lead to future implementation in medical programs. Our review aimed to give insight into the existing evidence, the gaps in the use of VR in medical education, and the potential benefits this modality of learning can have going forward in this field of study. Medical students have demonstrated significantly enhanced knowledge gain when using immersive interactive VR over screen-based learning. Given the improvements in students’ performance due to these dynamic and collaborative learning experiences, immersive VR training will become a standard in the development of clinical skills and ensure patient safety. Although the emphasis on empathy began later in the journey of gaining VR as a part of medical education, there is a need to gain those skills as early as possible in medical school. Implementing the use of VR as a supplement in medical education allows students to practice simulated patient encounters along with an array of different academic endeavors. By doing so, students will gain competency and confidence as they encounter patients during their clinical rotations and clinical practice.

## Introduction and background

Introduction

Virtual reality (VR) is a simulated experience achieved through the combination of physical hand devices and computer-generated software to create immersive and interactive environments, making artificial reality seemingly realistic. In order for an individual to experience VR, they must wear a headset that sets them in a three-dimensional environment that surrounds them. Depending on the type of VR system being used by the individual, tactile controllers are used to interact within the virtual environment. Users can then engage in the environment with objects, characters, and scenes, making individuals assume they are truly within the immersed environment. “This allows users to learn from experiences as they would in real life. This ability to deliver experiences on demand is where the power of VR lies.” [1] This review provides a comprehensive understanding of the progressive journey of VR, along with its current goals, advantages, disadvantages, and future outlook within medical education. By gathering information and providing analysis from various studies and systematic reviews, healthcare training programs such as schools and/or hospital systems can make evidence-based decisions on the utilization of VR as a learning platform.

Background 

VR is beginning to be adopted across medical and nursing fields with the objective of supplementing clinically relevant and practical teaching. The effectiveness of this interactive form of learning has come a long way with improvements in accessibility, cost, and technicalities. The simulated environment that VR has to offer began as screen-based learning, where the user interacts with a two-dimensional display. This form of VR requires very minimal setup time as it consists of haptic devices and a desktop screen [2]. Screen-based learning was previously considered "virtual reality" until the objective of immersive and interactive learning, using three-dimensional environments, was established as the standard in the VR experience. Using VR headsets to experience a sense of presence in a learning environment has now been integrated into such educational interventions, which sets this transformative approach apart from traditional learning [1]. In an effort to provide a complete picture of an environment, 360 video is a filming method where a student, while using headsets, can experience a setting from every direction, almost as if the student is in the middle of a film. Students can watch the video from different angles using a smartphone and VR goggles [3]. The drawback to this method is that it does not allow the student to actively engage with the environment through movement and interactions in a realistic manner, as 360 video captures only a linear recording. This passive experience disconnects the student from the immersive learning that VR is now designed to provide.

Both screen-based learning and 360-video have been effective stepping stones towards enabling the use of further advancing technology. Interactive VR offers an immersive and dynamic platform for medical training. These simulations benefit the learners by allowing them to interact within case scenarios and virtual wards, as well as with patients, colleagues, and relatives [1]. This method is commonly accomplished using VR head-mounted displays (HMDs), such as the Oculus Rift or HTC Vive [2]. As a means to mimic real-life encounters, the student can take a patient’s history and physical exam, investigate, diagnose, and provide treatment. In addition to the stressful and adaptive hospital environment, the patient is able to express emotions, concerns, and signs of a poor state of health. All of these factors play into a healthcare provider’s competency to think critically and clinically in decision-making. After each scenario, students receive debriefing and feedback on their performance from the system, which is a critical component in producing impactful, lasting improvements in learners. Such scenarios have now expanded across many medical fields, as companies such as Oxford Medical Simulation have implemented this educational tool globally [1]. This practice is now being used in many surgical programs and has proven to decrease injury, increase operation speed, and improve overall clinical outcomes. For example, when training for endoscopy surgery, the minimal set-up time and accessibility for repeated use make simulated VR systems successful in enhancing psychomotor technical skills [2]. Medical students have demonstrated significantly enhanced knowledge gain when using immersive interactive VR over screen-based learning. Although interactive VR simulations have become increasingly utilized in medical training, they do not replace the expert educator or clinical training but rather offer a supplemental and powerful educational tool. In addition, it is important to recognize that specific learning scenarios and objectives, such as abdominal palpation, can be better taught and practiced through physical simulation rather than VR simulation [1] (Table 1).

**Table 1:** Previous studies on the integration of virtual reality into established medical education programs

Study	System	Summary	Study or paper limitations
Using Virtual Reality in Medical Education to Teach Empathy [4]	Embodied Labs	The study developed pre- and post-assessments that were embedded within the software to assess the first-year students' outcomes. They accomplished this by integrating this assignment into their geriatric curriculum. The students were able to gain a first-person perspective on patients with age-related conditions such as hearing loss, vision loss, or Alzheimer's disease. Study results showed that VR enhanced students' understanding of age-related health problems and increased their empathy for older adults.	A more objective and valid assessment tool is needed to evaluate the outcomes of the intervention on student learning and empathy.
An Experimental Study on Usefulness of Virtual Reality 360 In Undergraduate Medical Education [3]	VR 360° videos	In addition to the positive experience and feedback received through the pre- and post-surveys, the undergraduate medical students' mean scores showed improvement in their knowledge retention, skills acquisition, and satisfaction levels. This study also emphasized using this technology to enhance communication with the entire healthcare team including patient, relatives, non-healthcare professions and multidisciplinary teams in healthcare.	This study design involved medical students who were in the same level, and the researchers stated that more well-rounded perspective may be obtained if medical students of different academic levels were included in the study. Also, this study concluded that more research needs to be completed to validate VR as a valuable education tool.
VR Simulation Leads to Enhanced Procedural Confidence for Surgical Trainees [6]	Hands on and passive learning methods for appendectomy and cholecystectomy	Participants were given assessments to test for knowledge acquisition after each intervention as well as post-surveys to gain the feedback from this learning experience. Traditional hands-on learning and passive learning have their own merits. Passive learning such as the use of videos should not be replaced by active, immersive learning such as VR. Rather, both can be used to to supplement one another as they both enhance different aspects of learning. The subjects showed greater confidence in their ability to reproduce the steps of the procedure and believed VR had greater utility as a learning strategy. On the other hand, the subjects believed the use of videos are easier to use and can complement the curriculum.	The study used a crossover study design rather than a case control study design, thereby increasing the risk of confounding variables in the study's results.
Virtual Reality and the Transformation of Medical Education [1]	VR Simulation Suites and VR Trolleys in Established Medical Education Institutions	This paper is a narrative review of how VR works, what it has to offer and its future in medical education. It was highlighted that the complexity in medical education is constantly changing. Therefore, having a tool such as VR can help accelerate clinical skills and competency, critical thinking, and patient safety. This paper states that the future of VR will have technological advances, become a more routine practice, and expand the use of multiplayer learners and inter professional roles.	This review briefly mentioned that the future of VR will emphasize humanistic skills early on in medical training but does not describe how it can accomplish this and why this is significant for medical education.

Interactive VR systems have a simple and quick setup, allowing learners to have a clinical scenario delivered to them within five minutes. This design benefits institutions by decreasing the amount of space and faculty time. Another advantage for the institutions is that VR allows simulations to be delivered at reduced costs and with fewer resources [1]. When comparing the time and costs involved in physical or in-person procedures, the VR system allows for more repetitive and accessible learning when taking finances and set-up time into account. These on-demand clinical experiences provide a learning environment that is more controllable, secure, and safe [3]. Currently, each VR kit ranges from $2,000 to $2,500, with costs continuing to trend down [4]. Due to the increased use of the VR system in various industries, there have been several studies that have highlighted the significance of integrating this simulated training into medical education [5].

## Review

Eight papers were included in this review. Four of the eight papers received a greater analysis due to their unique focuses and approaches on different aspects of VR's contribution to medical education. The studies were heterogeneous in terms of the design of interventions and the collection of data. The studies included are two randomized control trials, two with quasi-experimental designs, one crossover study design, and three non-experimental designs. The three non-experimental designs are literature reviews. Four electronic databases (PubMed, EMBASE, Cochrane, and ScienceDirect) were utilized. Studies with the terms “Virtual Reality,” “Virtual Reality Training,” “Empathy,” “Medical Student Simulation Training,” and “Behavioral Empathy” were eligible for inclusion.

Using virtual reality in medical education to teach empathy 

The University of New England, in its third year of using Embodied Labs’ VR system, studied the use of VR for training aging patients. The immersive experience placed users in the “shoes of the patient to teach about the aging experience from a first-person perspective.” Ultimately, the experiences included the first-person viewpoints of patients with macular degeneration, hearing loss, and Alzheimer’s disease. After assessment of the project implementation for the intended purpose, 178 first-year medical students demonstrated an increased understanding of empathy with geriatric patients after integrating this project into their geriatric curriculum with a total of six VR kits between two medical campuses. This study gained these results through pre- and post-assessments that were embedded in the software. Of note, there were some downfalls, including glitchy connections that have improved over time through software updates. Additionally, the study stated that a more objective assessment tool would be beneficial to accurately assess the improvements in student learning and empathy. Overall, the study concluded that VR immersion training is an effective teaching method to help develop empathy among medical and healthcare professional students [4].

An experimental study on usefulness of virtual reality 360 in undergraduate medical education 

A study conducted at the College of Medicine at King Saud bin Abdul-Aziz University for Health Sciences used 360-degree virtual reality immersion with experimental and control groups to study the effects of VR on effective communication with patients, relatives, non-healthcare professionals, and multidisciplinary teams in healthcare. A pre- and post-survey was completed by each participant, with a total of 169 medical students. Ultimately, the use of VR 360° showed a statistically significant improvement for undergraduate medical students in their knowledge retention, skills acquisition, and satisfaction levels by measuring their mean scores. Additionally, this study assessed the students' perceptions of using VR in medical education. 91% of the students agreed with the importance of technology integration in teaching and learning. 90% of the students believed technology integration would benefit them. 84% of students believed that using these tools appealed to a variety of learning styles. 70% of the students agreed that this technology encourages student-centered learning. 77% of students agree that virtual education will engage students in learning. 74% of students agreed that VR motivates students to learn. 69% of students believe VR will increase the quality of education by allowing students to learn at their own pace. Virtual education will increase the quality of education by allowing students to learn at their own pace. 61% of students believe VR can make a difficult concept easier. The drawbacks that students agreed to were that VR is expensive to implement (70%), and there are not enough concepts currently available to use VR (63%). This study highlighted that VR can also benefit interprofessional education by allowing undergraduate health professionals to learn and engage with each other's roles with the intent of improving collaboration and communication skills for better health care. Thus, it was concluded that ongoing research regarding VR simulation is needed to validate VR as an educational tool. This study design involved medical students who were at the same academic level, and the researchers stated that a more complete perspective may be obtained if medical students of different academic levels were included in the study [3]. 

VR simulation leads to enhanced procedural confidence for surgical trainees

A study at the University of Central Florida College of Medicine and the University of Florida College of Engineering studied the effects of active learning techniques using virtual reality to enhance the knowledge of medical students with limited hands-on operative experiences. They hypothesized that VR is a more effective modality for teaching laparoscopic surgical techniques to medical students than the more passive learning tools like videos. The study used two study groups; one group used a hands-on, active method such as VR, while the other used a passive video method when studying how to perform a laparoscopic appendectomy and cholecystectomy. Each intervention was followed up with a knowledge assessment. In addition, each student was given a post-survey to gain feedback and subjective interpretations of the learning experience. The results of the study showed that VR produced greater confidence in the ability to reproduce the steps of the procedure and had greater utility as a learning strategy than the video learning method. However, the subjects also believe that the passive video learning method is more simple to use and can serve as complementary to their medical curriculum. “Through this experiment, we evaluated a new learning tool for hands-on teaching of laparoscopic techniques when compared to traditional passive learning from a video. There are merits to both methods of learning and the greatest improvement in learning, confidence, and eventually performance may be the integration of the two instead of either individually." This study concluded that both forms of learning enhance different aspects of learning [6].

Virtual reality and the transformation of medical education

This white paper provides a narrative review of how VR stands in medical education today, what it has to offer, and its future in medical education. The paper explored two specific case studies. The first at the University of North Hampton created a VR simulation suite for nursing students to integrate VR scenarios with real-time peer contribution. The second study, at the University of Oxford, implemented mobile VR trolleys to transport the equipment wherever it was needed. This allowed medical students to share the virtual experience with others without faculty having to dedicate time to monitor students using the technology. In addition, this provided more student autonomy for a fully functioning VR system to be used at convenient hours. This paper emphasizes that the pace at which medicine is constantly changing in its complexity consequently requires adaptation in medical education. Therefore, in order to align with the advancements in medical knowledge and practice, VR serves as a tool to help accelerate a learner's ability to improve clinical skills and competency, critical thinking, and decision-making. This paper states that the future of VR will have technological advances such as better haptics and hand control. It also states that VR will be used more casually in practice and education, in which students and practitioners integrate VR into their routine training to further improve their performance. Lastly, it was emphasized that multiplayer VR will become increasingly available to allow different learners and interprofessional roles to interact in the same clinical scenario. This review briefly mentioned that the future of VR will be used to gain humanistic skills early on in medical training. However, the paper did not describe why this skill can influence the course of clinical training for students and how students can achieve this through VR learning [1].

Discussion

The reviews of the studies presented had multiple perspectives that were effective in evaluating the current state of VR being utilized in medical education. With the continuously evolving pace of medicine, medical education needs to keep up with the demand through modernization and technology, particularly in the simulation aspect. A complementary tool, such as VR, will enhance the ability to adapt to the demands of medical training in today's technologically advanced society by building clinical, technical, non-technical, and humanistic skills. The results that were presented in "VR Simulation Leads to Enhanced Procedural Confidence for Surgical Trainees" support that VR cannot replace traditional learning but rather supplement and enhance learning by combining VR with traditional learning to help solidify curriculum [6]. In the current VR experiences seen across different platforms, participants can make choices, implement their choices, and see the outcome. They can then react and respond to the choices they make, reducing the risk of adverse outcomes when doing so in real clinical situations.

VR simulation in medical education is projected to become a more common and casual practice where students have it incorporated into their routine. Given the improvements in students’ performance due to these collaborative learning experiences, it will become a standard in the development of clinical skills and increase patient safety during the clinical years. Looking forward, there will be more technological advances with future improvements in hand control, voice control, and haptics to better “blur” the lines between the real and virtual” [1]. Multiplayer VR systems are projected to become increasingly available, where learners can see and interact with one another and the patient in a virtual environment. As VR becomes increasingly available to the general public, it will allow more medical schools to acquire the technology and provide supplemental learning streams for students to solidify their skills using a multidisciplinary team approach, involving other healthcare professionals, patients, relatives, and non-healthcare professionals. This advancement will benefit interpersonal and professional skills by emphasizing humanistic care and teamwork as well as embodying real-life clinical experiences. The findings presented in "An Experimental Study on Usefulness of Virtual Reality 360 In Undergraduate Medical Education" support that VR enhances the student curriculum and provides another avenue to expand the multidisciplinary approach in medical school [3].

The development of empathy in healthcare professionals and students has become increasingly emphasized because, historically, empathetic behaviors have been shown to enhance patient satisfaction, patient compliance, and overall better care [7]. Traditionally, some may resort to practicing standardized patients (SPs), imagination, and/or watching videos as a way to gain the perspective of someone else; however, it can be cognitively taxing. While simulated experiences where the learner acts in the role of the patient can be effective in eliciting empathetic care, these standardized patient encounters have limitations such as lack of repeatability, increased time and cost, less variety in clinical scenarios and strategies, and a less safe learning environment. These factors are attributed to the fact that in-person standardized patient encounters require substantial resources, creating obstacles to independent, repeated practices for students and health caregivers. The disadvantages of the traditional approach highlight the need for another method that can enhance standardizability, repeatability, and safety for students while requiring fewer resources [7]. Therefore, perspective-taking through VR by reacting to the environment and gaining a sense of presence can reduce the amount of physical resources as well as mental resources expended on solely imagination. In addition, using VR to tailor to specific environments has been shown to enhance the reproducibility of the experience as well as reduce pre-existing biases that less immersive, traditional experiences have difficulty achieving. For example, past studies have compared the effectiveness of traditional perspective-taking and VR perspective-taking in efforts to gain empathy for the elderly, colorblind, schizophrenia, and dark-skinned populations and their experiences. All of this showed a more positive effect on empathy and prosocial behaviors while decreasing prejudice against out-group members in those using immersive VR experiences as opposed to traditional perspective-taking [8]. With these innovative uses of the VR system, there will be more opportunities to utilize this teaching method to help develop empathy early on in a medical student’s career.

Our review aimed to give insight into the existing evidence, barriers to VR in this field of study, and areas in which there are potential benefits for this platform progressing forward in the medical school curriculum. Compared to the multiple reviews and studies examined, this review focused on the incorporation of empathy in immersive training instead of only focusing on the technicalities and costs. Although some reviews briefly mention the significance of gaining empathy through VR simulation, they do not provide detailed explanations of how this can be accomplished. In addition, we have been able to identify areas of this VR in medical education that have less literature support, leading to areas of potential research. VR allows for the improvement of the competence of medical students, the emphasis on autonomy, and blended learning with multiple resources. As VR technology continues to improve and medical programs around the world implement it, it caters to the various learning styles of students and ultimately enhances the knowledge of interprofessional education as a whole.

## Conclusions

In efforts to incorporate VR training in medical education, it is important for the student to gain not only technical skills but also their humanistic skills. Although the emphasis on empathy began later in the journey of gaining VR as a part of medical education, there is a need to gain those skills as early as possible in medical school. With the limited clinical experiences provided in a student’s first and second years, this supplemental learning will provide each student with opportunities to improve their clinical skills. Implementing the use of VR as a supplement in didactic education allows students to practice simulated patient encounters along with an array of different academic endeavors. By doing so, students will gain competency and confidence as they encounter patients during clinical rotations and clinical practice.
